# HAPPi Kneecaps! Protocol for a participant- and assessor-blinded, randomised, parallel group feasibility trial of foot orthoses for adolescents with patellofemoral pain

**DOI:** 10.1186/s13047-020-00417-9

**Published:** 2020-08-02

**Authors:** Isobel C. O’Sullivan, Kay M. Crossley, Steven J. Kamper, Marienke van Middelkoop, Bill Vicenzino, Melinda M. Franettovich Smith, Hylton B. Menz, Anne J. Smith, Kylie Tucker, Karina T. O’Leary, Natalie J. Collins

**Affiliations:** 1 Division of Physiotherapy, School of Health and Rehabilitation Sciences, The University of Queensland, Brisbane, Queensland 4072 Australia; 2 La Trobe Sport and Exercise Medicine Research Centre, School of Allied Health, Human Services and Sport, College of Science, Health and Engineering, La Trobe University, Melbourne, Victoria Australia; 3 School of Public Health, University of Sydney, Camperdown, New South Wales Australia; 4 Department of General Practice, Erasmus MC University Medical Center, Rotterdam, The Netherlands; 5 Discipline of Podiatry, School of Allied Health, Human Services and Sport, College of Science, Health and Engineering, La Trobe University, Melbourne, Victoria Australia; 6 School of Physiotherapy and Exercise Science, Curtin University, Perth, Western Australia Australia; 7 School of Biomedical Sciences, The University of Queensland, Brisbane, Australia

**Keywords:** Patellofemoral pain, Adolescents, Feasibility, Foot orthoses

## Abstract

**Background:**

Patellofemoral pain (PFP) is a common cause of knee pain in adolescents, but there are limited evidence-based treatment options for this population. Foot orthoses can improve pain and function in adults with PFP, and may be effective for adolescents. The primary aim of this study is to determine the feasibility of conducting a full-scale randomised controlled trial (RCT) evaluating the effects of contoured foot orthoses on knee pain severity and patient-perceived global change, compared to flat shoe insoles, in adolescents with PFP. The secondary aim is to provide an estimate of treatment effects for foot orthoses, compared to flat insoles, in adolescents with PFP.

**Methods:**

This randomised, controlled, participant- and assessor-blinded, feasibility trial has two parallel groups. Forty adolescents (aged 12–18 years) with clinical symptoms of PFP will be recruited from Queensland, Australia. Participants will be randomised to receive either prefabricated contoured foot orthoses or flat shoe insoles. Both interventions will be fit by a physiotherapist, and worn for 3 months. Feasibility will be evaluated through assessing willingness of volunteers to enrol, number of eligible participants, recruitment rate, adherence with the study protocol, adverse effects, success of blinding, and drop-out rate. Secondary outcomes will evaluate knee-related pain, symptoms, function, quality of life, global rating of change, patient acceptable symptom state, and use of co-interventions, at 6 weeks and 3 months. Primary outcomes will be reported descriptively, while estimates of standard deviation and between-group differences (with 95% confidence intervals) will be reported for secondary outcomes.

**Discussion:**

Findings of this study will inform the feasibility of a full-scale RCT investigating the efficacy of contoured foot orthoses in adolescents with PFP. This full-scale study is necessary to improve the evidence base for management of adolescent PFP, and enhance outcomes for this population.

**Trial registration:**

ACTRN12619000957190.

## Background

Knee pain is responsible for considerable health impact in adolescents, with patellofemoral pain (PFP) one of the most common knee pain conditions affecting this population [1]. PFP affects almost 30% of adolescents [2], and can have substantial detrimental effects. These include reduced participation in sport and physical activity, which can have implications for general health, mental health and overall quality of life [3–5]. Paellofemoral pain in adolescents tends to be persistent [5], with one-quarter of adolescents experiencing ongoing PFP symptoms well into adulthood [6]. Together, this highlights the importance of optimal management of PFP in adolescents, to avoid ongoing symptoms and disability across the lifespan.

Despite the burden of adolescent PFP, very few studies have investigated treatments for this population. Only two randomised controlled trials (RCTs) have been conducted in adolescents with PFP, which evaluated exercise and patient education [7], and soft foot orthoses [8]. Due to the lack of direct evidence, clinicians involved in the management of adolescents with PFP tend to apply treatment guidelines based on evidence from adult studies [9]. However, adolescents are a unique population with their own physical and psychosocial characteristics [10] – they are not simply small adults. For example, adolescents with PFP do not demonstrate the same quadriceps and hip strength deficits as adults with PFP [10], especially in early adolescence (age 12–16 years) [11]. Evidence-based treatments evaluated in adult PFP cohorts may have different efficacy in adolescents with PFP. While successful outcomes with exercise therapy and multimodal physiotherapy (exercise combined with patellar taping and manual therapy) are observed in 62–81% of adults with PFP after 12 months [12, 13], only 38% of adolescents with PFP experience a successful outcome with a similar intervention [7, 10]. Adolescents with PFP also have lower success with exercise therapy (53%) compared to adults (67%) [10]. Poorer outcomes in adolescents could be attributable to lower adherence with home exercise programs [10]. There are a number of potential barriers to exercise adherence in adolescents, including competing priorities (e.g. school, sport and social commitments) and the high prevalence of bilateral PFP (which doubles exercise dose/time) [10].

Foot orthoses may be an effective intervention for adolescents with PFP. Worn bilaterally, foot orthoses have the potential to exert biomechanical and physiological effects on the lower limb during weight bearing activities, which typically aggravate PFP [14]. Foot orthoses are a recommended intervention for adults with PFP [9]. In our previous RCT, prefabricated contoured foot orthoses resulted in significantly greater global improvement than flat insoles over 6 weeks (number needed to treat 4, 95% CI 2 to 51) [12]. Compared to flat insoles, foot orthoses resulted in faster symptom improvement in adults with PFP, which is important given that symptom severity and duration are key determinants of long-term prognosis [15, 16]. Investigating the efficacy of foot orthoses in an adolescent population is needed to inform clinical guidelines specifically for adolescents with PFP.

Considering the expense and time required to run a full scale RCT, it is necessary to first determine feasibility. There has been only one previous RCT assessing the effects of foot orthoses in adolescents with PFP, published in 1993 [8]. The results demonstrated a greater reduction in PFP symptoms with soft foot orthoses and an exercise program, compared to flat shoe inserts and exercise. However, the results should be considered with caution given that the method of foot orthoses prescription does not reflect current clinical practice [17], that only females were included in the study cohort, and the small sample size (*n* = 20).

The primary objective of this study is to determine the feasibility of conducting a full-scale RCT evaluating effects of contoured, prefabricated foot orthoses on knee pain severity and patient-perceived global change, compared to flat insoles, in adolescents with PFP. The secondary objective is to provide an estimate of treatment effects for prefabricated foot orthoses, compared to flat insoles, in adolescents with PFP.

## Methods

### Experimental design

HAPPi Kneecaps! (sHoe inserts for Adolescents with Patellofemoral PaIn) is a randomised, controlled, participant- and assessor-blind, feasibility trial, with two parallel groups. The study design was developed in consultation with the SPIRIT 2013 statement [18] and the CONSORT 2010 statement extension to randomised pilot and feasibility trials [19]. Ethics approval was obtained through The University of Queensland’s Human Research Ethics Committee (HREC No. 2018000159). The trial was prospectively registered on the Australia New Zealand Clinical Trials Registry (ACTRN12619000957190; date registered 08/07/2019). Written informed consent will be obtained from all participants prior to participation in the study.

### Participants

Adolescent volunteers with a clinical diagnosis of PFP will be recruited from the community in Brisbane, Australia. We will use an active, targeted, comprehensive recruitment strategy across multiple platforms. This strategy will integrate successful methods from our previous patellofemoral RCTs [12, 20, 21], tailored to an adolescent population. The recruitment strategy will include: (i) *social media:* we will target key social media platforms used by adolescents and their parents, using free and paid advertising (e.g. Facebook, Twitter); (ii) *secondary schools (private/public) and sports clubs (*e.g. *netball, football, athletics)* will be provided with recruitment and educational resources on PFP; (iii) *community events:* we will target key events attended by adolescents and their parents (e.g. weekend markets, community fairs) with flyers and sandwich boards. We will also recruit from our existing databases of PFP volunteers, and through contacts with health clinics (e.g. general practitioners).

Sample size has not been formally calculated for this feasibility study. Based on previous work [22, 23], we estimated that 20 participants per group (*n* = 40) would allow observation of recruitment practicalities, acceptability and common adverse effects of the interventions, dropouts, and sample variability.

Male and female adolescent volunteers will be eligible for inclusion if they meet the following criteria: (i) aged 12–18 years; (ii) anterior knee pain of non-traumatic origin that is rated at least 3 on an 11-point numerical rating scale (0 = no pain, 10 = maximal pain); (iii) knee pain aggravated by activities that load the PFJ (e.g. squatting, stair climbing, running, jumping); (iv) knee pain present at some time during most weeks; and (v) knee pain of at least 2 months duration.

Volunteers will be excluded if they meet any of the following criteria: (i) concomitant pain at sites other than the anterior knee (e.g. other knee structures, hip, lumbar spine); (ii) history of knee, hip or spine surgery, or other suspected knee joint pathology (e.g. Osgood Schlatter’s Disease); (iii) planned lower limb surgery (e.g. arthroscopy); (iv) recent treatment for PFP (e.g. knee injections or physiotherapy within the previous 3 months; foot orthoses within the previous 12 months); or (v) any foot condition precluding the use of foot orthoses.

### Study procedures

**Fig. 1:**
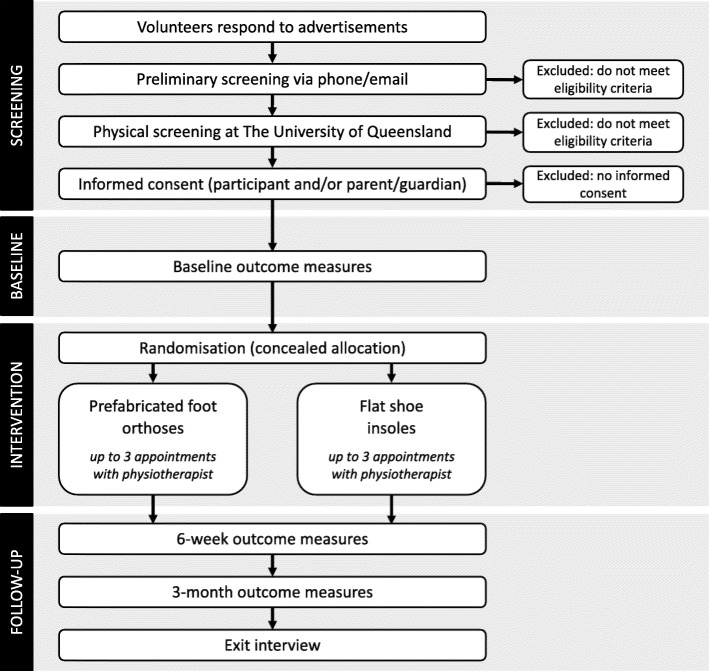
Participant flow through the study

After undergoing preliminary screening for eligibility criteria via email and/or telephone, participants will be invited to undergo physical screening at The University of Queensland to confirm the presence of PFP, and absence of other anterior knee pain conditions (e.g. Osgood Schlatter’s Disease, patellar tendinopathy) (Fig. 1). Eligible volunteers (and/or their parent/guardian) will then provide written informed consent prior to collection of baseline measures. All screening procedures and baseline data collection will be performed by a registered physiotherapist (ICO), from whom group allocation will be concealed.

Participants will be randomised to one of two interventions: (i) prefabricated contoured foot orthoses, or (ii) flat shoe insoles. Randomisation procedures will be via concealed allocation, with the randomisation sequence held by an offsite investigator (KTO). Due to the nature of the two shoe insert interventions (contoured vs. flat inserts), it is not possible for study physiotherapists to be blinded to group allocation. Primary outcomes for feasibility will be collected by a blinded assessor (ICO). Secondary outcomes will be self-reported by participants, who are considered assessors. To facilitate blinding of participants (and therefore assessors of secondary outcomes) via limited disclosure, participants will be informed that they will be randomised to one of two shoe insert interventions, and will not be informed of the differences between the two inserts or the study hypotheses [12, 20].

### Interventions

The interventions will be fit by a registered physiotherapist working in a private Physiotherapy clinic in the greater Brisbane region. Study physiotherapists will be trained in fitting procedures, which will follow established algorithms [20]. Participants will receive up to four pairs of inserts fit to their daily shoes (e.g. school shoes, sports shoes, casual shoes). The inserts will be fitted to the participants’ shoes that accommodate foot orthoses and provide the best support. Fitting procedures for both the contoured and flat shoe inserts will be based on comfort, to maximise wear time and potential therapeutic effects. Participants will attend up to three appointments with the study physiotherapist to ensure adequate comfort of the inserts, and will receive written instructions for using and adapting to the inserts. Participants will be asked to wear their inserts as much as possible during their waking hours, and encouraged to transfer the inserts between different shoes as required to maximise wear time. This is consistent with current clinical practice. Participants in both groups will also receive general information and advice about PFP and activity in a participant handbook (see Additional file 1).

#### Prefabricated contoured foot orthoses

**Fig. 2:**
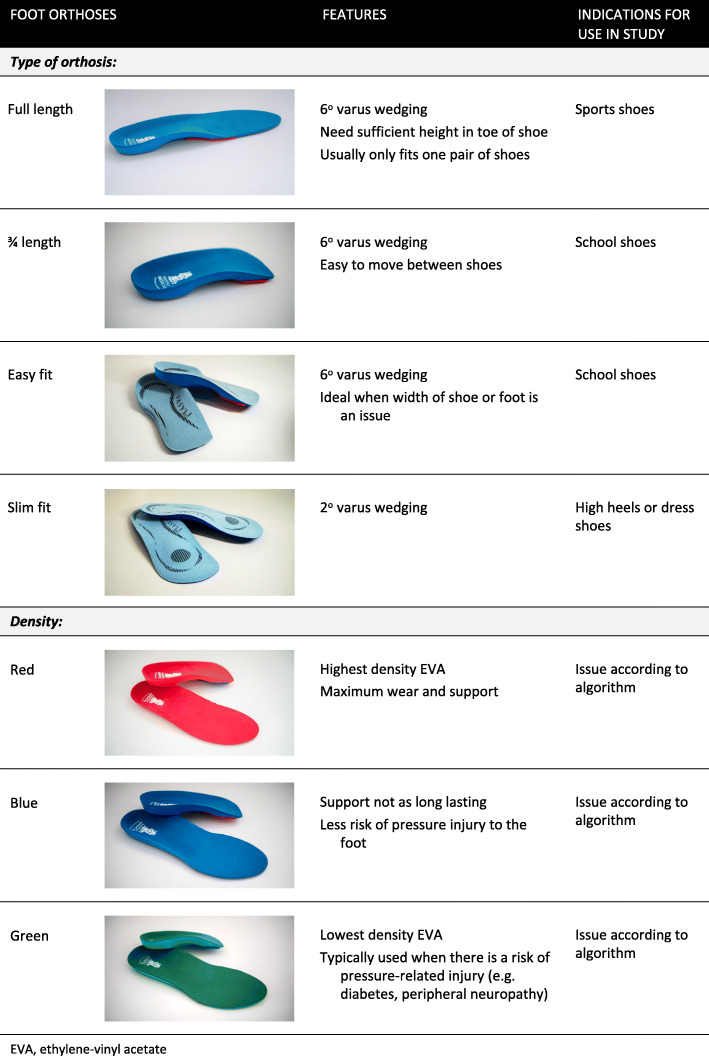
Prefabricated contoured foot orthoses

Participants randomised to the contoured foot orthoses group will receive commercially available prefabricated foot orthoses (Vasyli Medical, Labrador, Australia) (Fig. 2). These will be from the same range as the foot orthoses prescribed in our previous RCT in adults with PFP [12]. These orthoses are manufactured from ethylene-vinyl acetate (EVA) with options of a high (hard, Shore A 70°), medium (Shore A 55°) and low (soft, Shore A 45°) density, and have an inbuilt arch support and varus wedging. Modifications will be made to the foot orthoses to achieve a comfortable fit, via the addition of wedges or heat moulding as per our published algorithm [20].

#### Flat shoe insoles

**Fig. 3:**
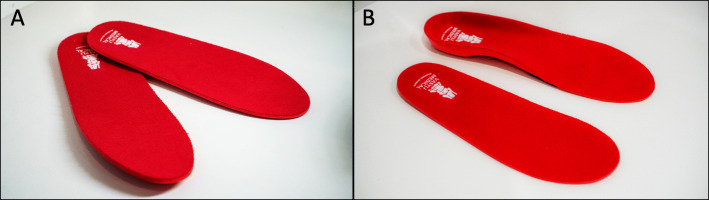
Flat shoe insoles (**a**), and image showing the difference in arch contouring and wedging between the flat insoles and prefabricated contoured foot orthoses (**b**)

Participants randomised to this group will receive flat shoe inserts manufactured from high density EVA (Fig. 3). The flat insoles will be of 3 mm uniform thickness along their length, and have an identical covering fabric as the contoured foot orthoses. This is to control for any potential effects of the arch contour and wedging associated with the contoured foot orthoses. As in our previous RCT, the flat insoles will be described to participants as an intervention to enhance sensory feedback [20]. Following our fitting algorithm [20], the flat insoles will be heat moulded to enhance comfort as indicated.

### Adverse events

If participants experience any adverse events during the study period (e.g. excessive pressure from the shoe inserts, blistering, increase in knee pain, onset of new pain elsewhere), they will be encouraged to report these to the investigator and/or their study physiotherapist. In this event, standard clinical practice principles will be implemented. As required, participants will attend an additional appointment with their study physiotherapist. The intervention will be modified for comfort according to procedures described above. If necessary, the shoe inserts will be removed until pain settles and slowly re-introduced as the participant tolerates. Such events will be recorded as an adverse event, and participants will be followed up at regular intervals to monitor changes in symptoms and to ensure they are progressing sufficiently. If symptoms associated with the adverse event are unable to be resolved, the intervention will be ceased.

### Concomitant care

If participants are taking regular medications (e.g. anti-inflammatories), they will be permitted to continue this throughout the study duration. If at any point during the study they wish to increase or start taking pain management medication, participants will be encouraged to report this to the investigator (ICO). At the time of entry into the study, participants will be asked refrain from commencing new treatments for their knee pain for the duration of the study (e.g. therapeutic exercise), and to avoid using other assistive devices such as knee braces or other footwear interventions for the study duration. Participants will be asked to report any use of co-interventions in log books (see Additional file 2). If at any stage participants are not satisfied with their allocated intervention, they will be encouraged to report this to the investigator (ICO) for appropriate management.

### Outcome assessment

At baseline, demographic and PFP data including age, sex, weight, height, affected knee, bilaterality of PFP, symptom duration, and aggravating activities will be collected. Participants will complete patient-reported outcome measures at baseline, and at 6 weeks and 3 months post-randomisation. Data collection will largely be completed via an online platform (REDCap), but participants will have the option to complete a paper version. For the study duration, participants will be asked to keep a daily log (assisted by their parent/guardian as required) of: activities or sport completed each day, knee pain severity (rated on a 10-point numerical rating scale; 0 = no pain, 10 = worst pain imaginable), type of shoes worn that day, hours spent wearing the footwear and shoe inserts, adverse effects associated with the shoe inserts, medication use, and any other comments (see Additional file 2). Study physiotherapists will record attendance, prescription notes and adverse events during fitting and follow-up. The primary endpoint is 3 months.

#### Primary outcomes

The primary outcome is the feasibility of conducting a full-scale RCT. Feasibility will be assessed by evaluating the following outcomes.

1. Willingness of participants to enroll in the study (from recruitment database).
2. Number of eligible volunteers (from recruitment database).
3. Recruitment rate (from recruitment database).
4. Adherence with allocated intervention and log book completion (from Study Practitioner notes, and participant log book).
5. Adverse events (from Study Practitioner notes, adverse events database and participant log book).
6. Success of blinding (risk of performance and detection bias) (from *Credibility and Expectancy Questionnaire*).
7. Drop-out rate (from trial database).

The following parameters were set to inform feasibility: (i) a recruitment rate of 1 participant per week; (ii) minimum adherence with shoe insert wear of 2 h per day, 5 days a week; and (iii) a drop-out rate of ≤20%.

Credibility and the participant’s expectations of treatment will be evaluated using the Credibility and Expectancy Questionnaire [24]. This will be completed at baseline, and at the participant’s second visit with the study physiotherapist (~ 2 weeks post-randomisation). The Credibility and Expectancy Questionnaire consists of six items organised into two sections; four items related to thoughts, and two items related to feelings. A subsequent study determined that credibility is derived from the first three thought items, and expectancy derived from the fourth thought question and the two feeling questions [25]. The Credibility and Expectancy Questionnaire has been administered in adolescent populations in previous studies [26, 27].

#### Secondary outcomes

Secondary outcomes will be collected at baseline, 6 weeks and 3 months.

##### Knee pain severity

Participants will be asked to report their usual and worst pain in the preceding week, as well as pain with a self-nominated aggravating activity. Pain severity will be measured using 100 mm visual analogue scales (where 0 mm = no pain and 100 mm = worse pain imaginable), which have established reliability and validity for PFP [28].

##### Knee injury and Osteoarthritis Outcome Score Child Version (KOOS-Child)

The KOOS-Child assesses five subscales: pain (9 items), symptoms (7 items), difficulty during daily activities (17 items), ability to participate in sport and recreation (7 items), and knee related quality of life (QoL) (6 items) [29]. Participants respond to each question using 5-point Likert scales from 0 (no problems) to 4 (extreme problems). Raw scores are transformed to a 0–100 scale, with 0 representing no knee problems and 100 representing extreme knee problems. KOOS-Child demonstrates good measurement properties in adolescents with knee conditions [30].

##### Knee injury and Osteoarthritis Outcome Score – Patellofemoral subscale (KOOS-PF)

Along with KOOS-Child, participants will complete the KOOS-PF. This subscale was developed for use in people with PFP and patellofemoral osteoarthritis, and designed to be used in conjunction with the five original KOOS subscales [31]. KOOS-PF has 11 items, with identical response and scoring parameters as KOOS-Child (described above). KOOS-PF has adequate measurement properties in adults with PFP [31], although these have not yet been evaluated in adolescents.

##### Global rating of change (GROC)

A 7-point Likert scale will be used to assess GROC at 6 weeks and 12 weeks. Participants will be asked to respond to the question *‘Overall, how has your knee pain changed since the start of the study?’*, using the following responses: ‘completely recovered’, ‘strongly recovered’, ‘slightly recovered’, ‘same’, ‘slightly worse’, ‘much worse’, and ‘worse than ever’. GROC has been used in a previous RCT of adolescents with PFP [7].

##### Patient acceptable symptom state (PASS)

The PASS evaluates the maximum level of symptoms, beyond which the participant considers themselves to be well [32]. Participants will be asked to respond either ‘yes’ or ‘no’ to the following question: *‘Considering all the activities that you do in your daily life, how well you can do these activities, and your level of pain, do you think that your current state is satisfactory?’*

##### Anterior knee pain scale (AKPS)

The AKPS contains 13 items related to current knee symptoms and function [33]. Each item’s response is weighted and summed to produce an overall score between 0 and 100, where 0 represents maximal disability and 100 represents no disability. The AKPS is widely used in studies on PFP, and has established reliability and validity in adults with PFP [28].

##### Youth quality of life – short form (YQOL-SF)

The YQOL-SF is a generic assessment of quality of life in individuals aged 11–18 years with and without chronic disease or disability [34]. YQoL-SF version 2 consists of 15 items derived from the perceptual instrument of the YQOL-R, and assess aspects of sense of self, social relationships, environment and general quality of life. Participants respond to each item on a scale from 0 (not at all) to 10 (completely or a great deal). Overall transformed scores range from 0 to 100, where the higher score represents better quality of life.

##### EuroQol-5D-5L

The EQ-5D (EuroQoL) questionnaire is a generic measure of health-related quality of life, comprised of five items covering five dimensions: (i) mobility; (ii) self-care; (iii) usual activities; (iv) pain/discomfort; and (v) anxiety/depression [35]. Participants will complete the 5 L version, where each subscale has five possible responses. Participants will also complete a visual analogue scale for self-reported health state, where 0 represents worst imaginable health state and 100 represents the best imaginable health state. We elected to use the adult version of EQ-5D-5L for this study, rather than the youth version, as some of our participants will fall outside the recommended age group for the EQ-5D-Y (age 8–15 years), and to facilitate comparison with data collected in previous studies in adolescents with PFP [7].

##### Use of co-interventions

Participants will be asked to keep a daily record of any use of co-interventions, outside their allocated intervention, utilized throughout the study (e.g. pain medication, physiotherapy, knee brace, other footwear interventions). This will be recorded in their log books over the three-month study period.

### Data management

Each participant’s information will be coded in a re-identifiable format, and stored in a database with no group identifier to maintain blinding of the investigator. Electronic databases containing participant contact details will be password protected. All databases will be stored on a password protected computer. All hard copies of data will be coded and stored in a locked filing cabinet in the School of Health and Rehabilitation Sciences at The University of Queensland.

### Planned statistical analyses

Descriptive statistics will be calculated for the primary feasibility outcomes. Estimates of i) standard deviation of the secondary clinical outcomes, and ii) between-group differences in secondary clinical outcomes, with accompanying 95% confidence intervals, will be calculated.

### Post-trial care

If participants experience any adverse events after completion of the study, they will be referred for follow up by appropriate health care providers. Participants will be permitted to keep their intervention after completion. On request, participants will be provided with additional shoe inserts on completion of the study (either contoured or flat shoe inserts). The contact details of the primary investigator will be available to each participant for any concerns related to the study.

### Study exit interview

After study completion, participants will be invited to partake in a one-on-one semi-structured exit interview. Questions will be aimed at further exploring aspects of study feasibility, and credibility and acceptability of the interventions, and burden of assessment schedule. Interviews will be conducted by the blinded investigator (ICO).

### Distribution of results

Once completed, study outcomes will be made available to participants via email or post on request. Outcomes will be disseminated via peer-reviewed publications, and submission of abstracts to appropriate national and international conferences.

## Discussion

PFP in adolescents is a problem, with clear implications for function, quality of life, and future knee health. Current clinical guidelines for PFP management are largely based on studies conducted in adults with PFP, with minimal data available regarding efficacious treatments for adolescents with PFP. Given the significance and potential impact of PFP in adolescents, it is imperative that we identify interventions that are acceptable and effective for this specific population. This feasibility study is the first step in evaluating foot orthoses as an intervention for adolescents with PFP.

We chose to evaluate prefabricated foot orthoses rather than custom foot orthoses in adolescents with PFP. This was based on the greater accessibility and substantially lower costs associated with prefabricated orthoses compared to custom orthoses [36]. Eng & Pierrynowski [8] evaluated the effect of medially posted soft foot orthoses in 20 adolescent females with bilateral PFP (mean ± SD age 15 ± 1 years, range 13–17 years). Participants were randomized to either posted or flat insoles, with both groups receiving an exercise program. After 8 weeks, the group who received medially posted orthoses had significantly greater improvements in pain severity during running, stair ambulation and squatting, compared to the group who received flat insoles. Notably, significant between-group differences at 8 weeks tended to exceed the minimal clinically important difference for pain measured on a visual analogue scale (2 cm) [28]. While this study provides important preliminary information regarding the potential effects of foot orthoses for adolescents with PFP, there have been no further RCTs in this area since 1993. Furthermore, the soft foot orthosis prescription used in the study by Eng and Pierrynowski [8] likely does not reflect current prescription practices [17]. Together, this highlights the need for further studies in order to support the use of foot orthoses in adolescents with PFP.

Many adolescents experience bilateral symptoms (70% versus 43% in adults) [37]. Foot orthoses are typically worn bilaterally, and thus have potential effects on both lower limbs during weight bearing activities. This is important for PFP, in that most aggravating activities involve weight bearing. Furthermore, because foot orthoses can be worn during physical activity and exercise (including therapeutic exercise), they may play a role in relieving pain and facilitating participation. Importantly, the use of a passive intervention such as foot orthoses may help to overcome issues with treatment adherence, which can be a substantial barrier in adolescents. Foot orthoses require minimal time or cost in that they are worn in everyday footwear, and easily transferred from school shoes to sports shoes. They have minimal side effects (e.g. rubbing and blistering), and in the case that they do, can be adjusted to enhance comfort [12].

This feasibility study is an important initial step in evaluating the efficacy of foot orthoses for adolescents with PFP, and has a number of strengths. The study design adheres to the SPIRIT guidelines [18] and CONSORT extension for pilot and feasibility studies [19]. Key features of the study design include randomisation, concealed allocation, and blinded data collection and analysis. The interventions to be evaluated in this study are simple to replicate in clinical practice, and are being administered in the clinical setting by physiotherapists. Secondary outcome measures that will provide estimates of treatment effects have been selected based on their applicability for use in adolescents, as well as their established measurement properties in PFP and, where possible, adolescents.

## Conclusion

HAPPi Kneecaps! will determine the feasibility of conducting a full-scale RCT evaluating the effects of foot orthoses on knee pain severity and patient-perceived global change, compared to flat insoles, in adolescents with PFP. Secondary outcomes will provide estimates of treatment effect sizes for prefabricated foot orthoses, compared to flat insoles, in this population. Given the high prevalence and burden of PFP in adolescence, and potential long-term sequelae, this study is an important first step in identifying appropriate and efficacious interventions for this population.

## Supplementary information

**Additional file 1.**

**Additional file 2.**

## Data Availability

De-identified individual participant data will be collected during the trial. Access to this data will be determined on a case-by-case basis at the discretion of the Principal Investigator, after publication of the study, with a requirement to sign a data access agreement.

## Abbreviations

AKPS: Anterior Knee Pain Scale
CONSORT: Consolidated Standards of Reporting Trials
EQ-5D: EuroQol-5D
EVA: Ethylene-vinyl acetate
GROC: Global Rating of Change
KOOS: Knee injury and Osteoarthritis Outcome Score
KOOS-PF: Knee injury and Osteoarthritis Outcome Score – Patellofemoral Subscale
PASS: Patient Acceptable Symptom State
PFP: Patellofemoral pain
RCT: Randomised controlled trial
SPIRIT: Standard Protocol Items: Recommendations for Interventional Trials
YQoL-SF: Youth Quality of Life – Short Form
