# Supplementary material for: HAPPi Kneecaps! Protocol for a participant- and assessor-blinded, randomised, parallel group feasibility trial of foot orthoses for adolescents with patellofemoral pain
Source: J Foot Ankle Res. 2020 Aug 2;13:50. doi: 10.1186/s13047-020-00417-9 (PMC7397622; doi:10.1186/s13047-020-00417-9)
Supplement: Supplementary file 1 — Additional file 1. [file 13047_2020_417_MOESM1_ESM.pdf]

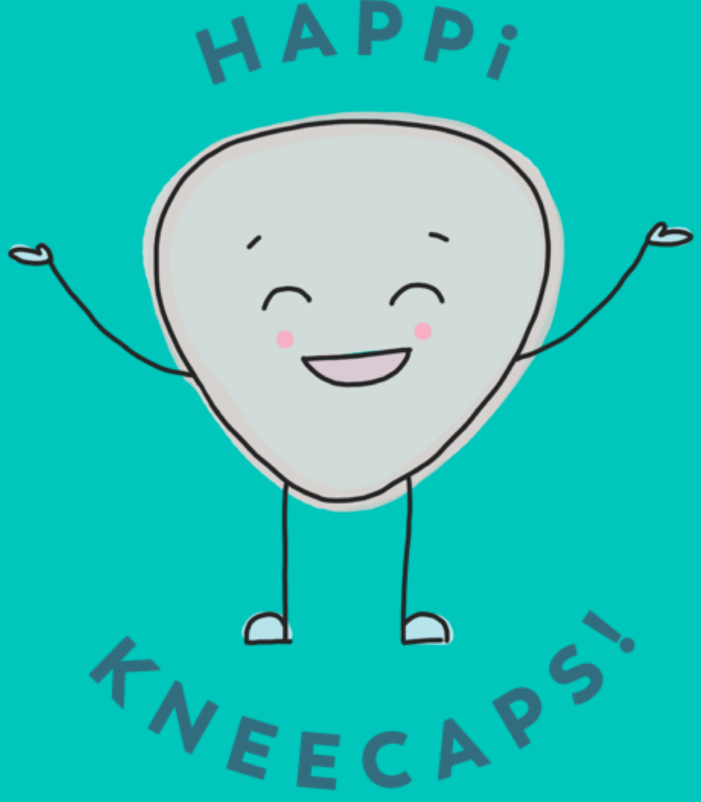

HAPPi Kneecaps!

A feasibility trial of sHoe inserts for Adolescents with Patellofemoral Pain

Participant Handbook

Arthritis AUSTRALIA

The University Of Queensland AUSTRALIA
CREATE CHANGE

What's in this handbook?

1. Information about kneecap pain & shoe inserts .... page 3
2. So I'm in the study… what happens now? .... page 4
3. How can I get the most benefit from my shoe inserts? .... page 5
4. Some common questions about HAPPi Kneecaps! .... page 7
5. Meet the HAPPi Kneecaps! Study research team .... page 8
6. Contact information .... page 9

HAPPi KNEECAPS!

1. Information about kneecap pain & shoe inserts

What is kneecap pain and why is it a problem?

Did you know that 1 in every 3 adolescents has knee pain? Knee pain can affect many aspects of your life, including your ability to be active or play sport, participate in school activities or hobbies, and hang out with your friends. We know that knee pain is the main reason why adolescents stop or decrease their sport participation… we want to try to stop this by finding treatments that can help knee pain!

Pain around the kneecap (kneecap pain) is the most common type of knee pain that adolescents, such as you, experience. Kneecap pain is typically located at the front of your knee (around your kneecap). Activities that load the kneecap – such as squatting, going up and down stairs, and running – tend to make the pain worse.

We also know that kneecap pain doesn't go away by itself. It's really important that we find treatments that can help kneecap pain… that's where HAPPi Kneecaps! comes in!

Shoe inserts might be the solution!

We are testing a simple treatment that is worn in your shoes… shoe inserts! Early information from researchers suggests that shoe inserts can help with normal movement and shock absorption of your knee during the activities that make your knee sore. Of course, shoe inserts are only effective while they are being worn in your shoes.

Shoe inserts are easy to fit to your everyday shoes, such as your school shoes, sports shoes and casual shoes. Once they are inside your shoes, no one can tell that they are there. The benefit of this treatment is that once the inserts are fit to your shoes, you don't have to do anything else, and the inserts will do their job while you go about your day. It's that simple!

2. So I'm in the study… what happens now?

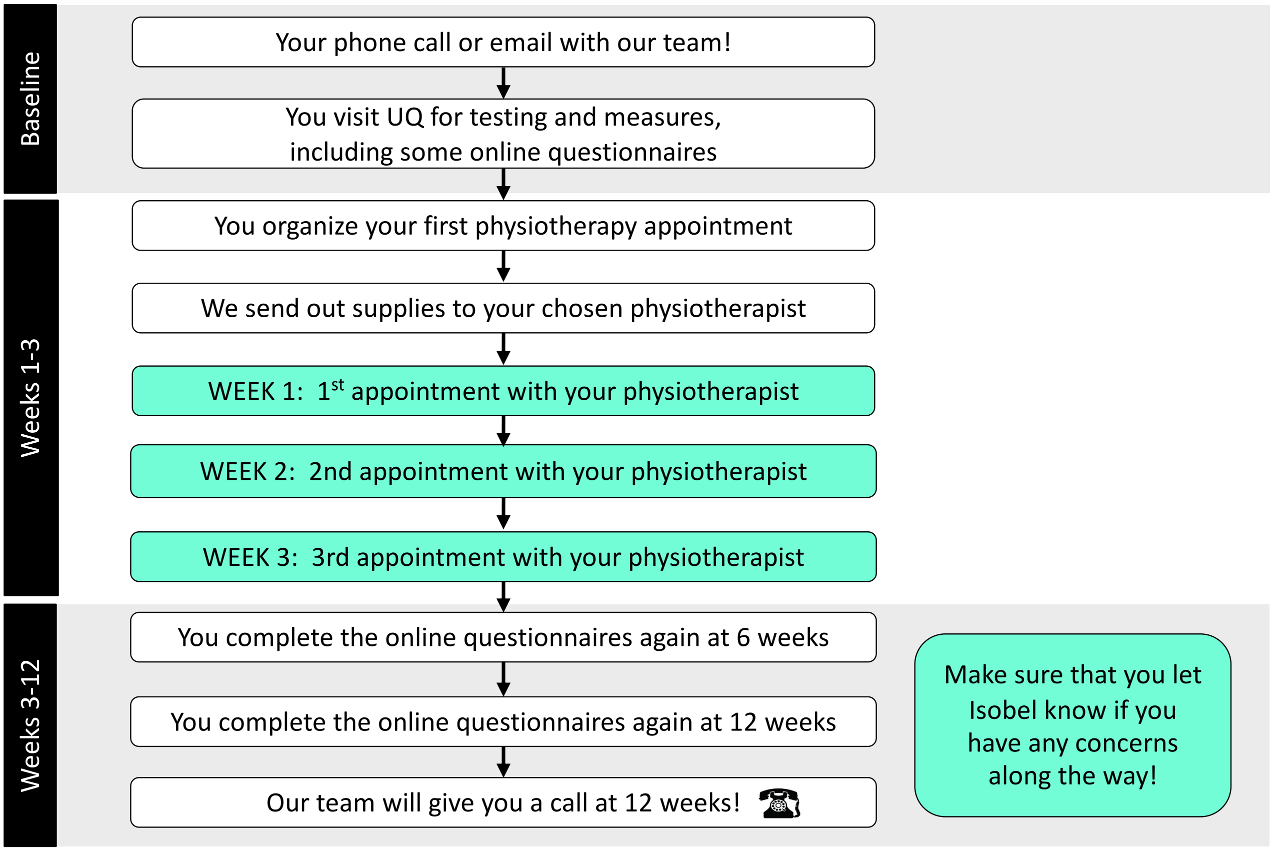

Your first appointment with your HAPPi Kneecaps! physiotherapist will last about 30 minutes. They will fit your shoe inserts into shoes that you wear most often. ***Don't forget to take the shoes that you wear most often to your first appointment!***

Your second appointment will be about one week later. Your physiotherapist will assess how your shoe inserts are going, and fit some more shoe inserts to other shoes that you commonly wear. It is important you attend this second appointment and inform your HAPPi Kneecaps! Physiotherapist how the shoe inserts are going.

Your third appointment will be similar to the second appointment. You will have a chance to talk to your HAPPi Kneecaps! Physiotherapist about any ongoing issues that you are having with the shoe inserts. After the third appointment, you should be comfortable and HAPPi in all the shoe inserts that your Physiotherapist has given you. ***At each appointment, it's really important that you tell your Physiotherapist if your shoe inserts are not comfortable.***

3. How can I get the most benefit from my shoe inserts?

As a participant in the HAPPi Kneecaps! Study, you will be provided with shoe inserts to wear in your everyday shoes and your sports shoes. The shoe inserts will be fitted by your physiotherapist using a special procedure.

The most important thing about your shoe insert treatment is that you wear them!

Remember that:

- The shoe inserts will only have an effect on your knee pain if you wear them.
- To gain relief from your knee pain, you need to wear the shoe insert as much as possible throughout the day.
- This includes when you are at school, at home, going out, and when exercising.

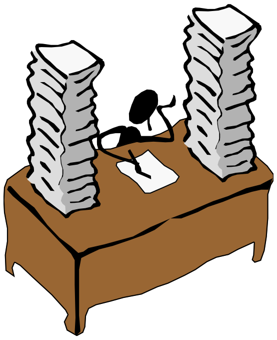

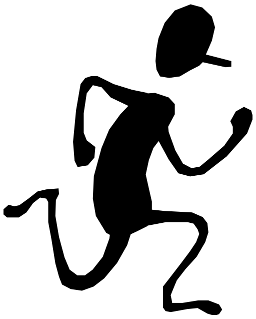

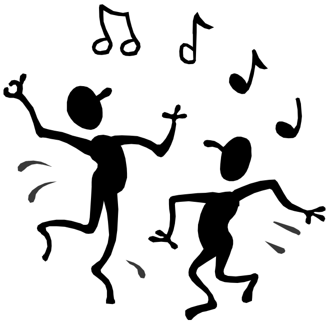

When the shoe inserts are in your shoes, you may notice a little pressure under the sole of your foot. It is essential, though, that the pressure is comfortable. If the shoe inserts are not comfortable, they will not have their desired effect. Your physiotherapist is aware of the importance of comfort, and will modify the shoe inserts using various techniques until they are comfortable.

Initially, the shoe insert may feel unfamiliar in your shoes. This is because your body is adjusting to the new position and movement of your foot and leg. This sensation should disappear after a short time as your body adapts. If it lasts longer than two weeks, please let your physiotherapist know.

Because you will have measures taken at 6 weeks and 12 weeks, it is important that you keep wearing the shoe insert for the entire 12-week period. This will give you the maximum benefit from the shoe inserts, and give us the most accurate information about how useful they are for treating kneecap pain.

If at any time you have any problems with the shoe inserts, please contact your physiotherapist as soon as possible.

Wearing your shoe inserts: a summary

You should:

- Have all shoes that you commonly wear fitted with the shoe inserts
- Wear shoes with shoe inserts for the entire day
- Avoid going barefoot
- Wear the shoe inserts continuously for the entire 12 weeks of the study
- Tell your physiotherapist if the shoe inserts are not comfortable

4. Some common questions about HAPPi Kneecaps!

Can I have other treatment during the study?

We ask that, where possible, you do not have any other forms of treatment during your time in the HAPPi Kneecaps! Study. This might include treatments such as physiotherapy, fancy tingly machines, natural therapies, or injections into your knee.

If you need to take any medication for your knee pain during the study (e.g. Panadol, Nurofen), we ask that you please record this in your logbook each day.

I have pain in both knees… will both of my knees be treated in this study?

Your shoe inserts will be fit to your left and right shoes. This is to make sure that they are comfortable (and that you don't walk funny!). It also means that, if you have pain in both knees, then both of your knees will receive the treatment. We will choose one of your knees to be your 'study knee'. When you complete your questionnaires, just make sure that your responses relate to your study knee.

Can I keep playing sport during HAPPi Kneecaps!?

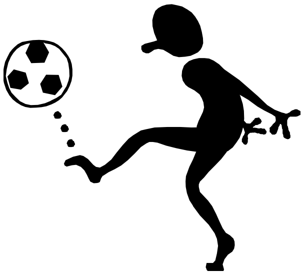

Absolutely! It's important that you keep being physically active. We encourage you to continue your normal activities for the duration of the study, provided that the shoe inserts are worn during the activity. However, sporting activities that make your knee pain worse may need to be modified. A good rule of thumb is to make sure that the activity does not cause you pain above 5 out of 10 during the activity, or make your knee pain worse the next day. If in doubt, please contact your HAPPi Kneecaps! physiotherapist.

If you experience severe pain (more than 5 out of 10) or swelling during an activity, you should stop the activity immediately and contact your HAPPi Kneecaps! physiotherapist.

Who should I contact if I have any problems or questions?

For questions about your shoe inserts, please contact your HAPPi Kneecaps! physiotherapist.

If you have any general questions about the study, please contact Isobel O'Sullivan on email isobel.osullivan@uq.edu.au or phone xxxx xxx xxx.

5. Meet the HAPPi Kneecaps! team

Dr Natalie Collins

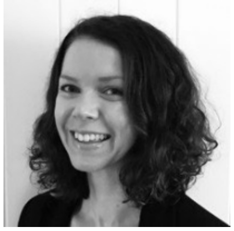

Physiotherapist,
Lecturer in the School of Health and Rehabilitation Sciences at The University of Queensland
http://researchers.uq.edu.au/researcher/12040

Isobel O'Sullivan

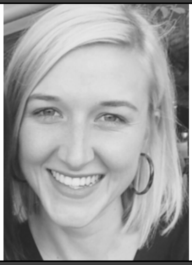

Physiotherapist, PhD Candidate, School of Health and Rehabilitations Sciences at The University of Queensland
https://shrs.uq.edu.au/profile/3399/isobel-osullivan

A/Prof Steve Kamper

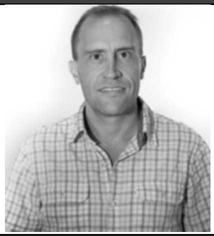

Associate Professor in the School of Public Health at the University of Sydney
http://sydney.edu.au/public-health/people/academics/profiles/steven.kamper.php

Prof Kay Crossley

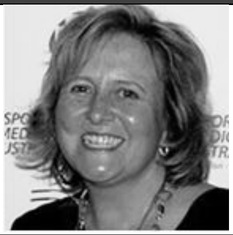

Physiotherapist, director of the La Trobe University Sport and Exercise Medicine Research Centre (LASEM)
http://www.latrobe.edu.au/school-allied-health/about/our-staff/profile?uname=KCrossley

A/Prof Marienke van Middlekoop

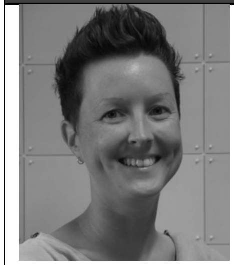

Assistant Professor at the Department of General Practice at the Erasmus MC Medical University in Rotterdam, the Netherlands
https://www6.erasmusmc.nl/huisartsgeneeskunde/research/employees/seniorstaff/6922330/?lang=en

Prof Anne Smith

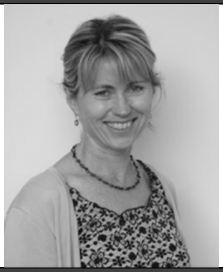

Physiotherapist, Professor in the School of Physiotherapy and Exercise Science at Curtin University
https://staffportal.curtin.edu.au/staff/profile/view/Anne.Smith/

Prof Bill Vicenzino

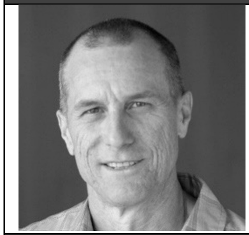

Physiotherapist and Professor in the School of Health and Rehabilitation Sciences at The University of Queensland
http://researchers.uq.edu.au/researcher/53

Dr Kylie Tucker

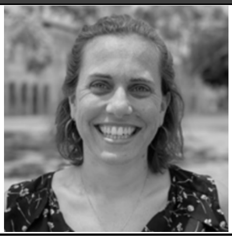

Senior Lecturer in the School of Biomedical Sciences Faculty of Medicine at The University of Queensland
https://researchers.uq.edu.au/researcher/1745

Dr Melinda Smith

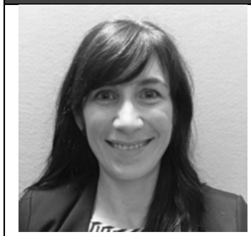

Physiotherapist and UQ Development Fellow in the School of Health and Rehabilitation Sciences at The University of Queensland
https://researchers.uq.edu.au/researcher/21292

6. Contact information

Your physiotherapist is:

Name: ____

Clinic: ____

Address: ____

Phone number: ____

If you have any questions or concerns about your shoe inserts, please contact your physiotherapist.

If you have any general questions about the study, please contact:

Isobel O'Sullivan
Physiotherapist, PhD Candidate
The University of Queensland
Email: isobel.osullivan@uq.edu.au

Thank you for your participation!
