# Supplementary material for: HAPPi Kneecaps! Protocol for a participant- and assessor-blinded, randomised, parallel group feasibility trial of foot orthoses for adolescents with patellofemoral pain
Source: J Foot Ankle Res. 2020 Aug 2;13:50. doi: 10.1186/s13047-020-00417-9 (PMC7397622; doi:10.1186/s13047-020-00417-9)
Supplement: Supplementary file 2 — Additional file 2. [file 13047_2020_417_MOESM2_ESM.pdf]

HAPPi Kneecaps!
Daily diary

Name: ____

Participant ID: ______________________________________

KNEE PAIN SCALE:

Please use the scale below to rate your knee pain is each day, and record it in the tables on the following pages. The faces and descriptions will help you to rate your pain each day. For example, 0 means that you have no pain, while 10 means that your knee hurts the worst that you could imagine. A pain rating of 5 indicates a moderate amount of pain.

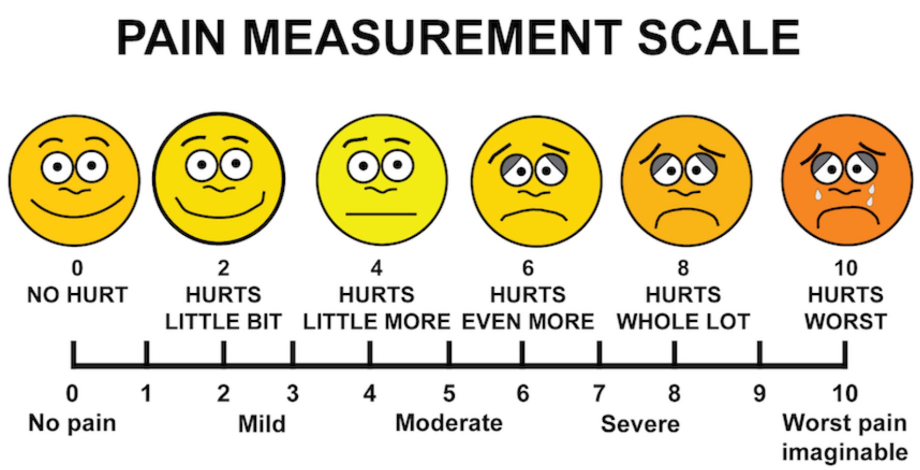

If you have any questions about HAPPi Kneecaps!, please contact:

Isobel O'Sullivan

PhD Candidate, School of Health and Rehabilitation Sciences

The University of Queensland

Email: isobel.osullivan@uq.edu.au

FOOTWEAR TYPES:

Please use the descriptions below to record which shoes you wore each day. If your shoes don't match any of the options below, you can provide a brief description instead.

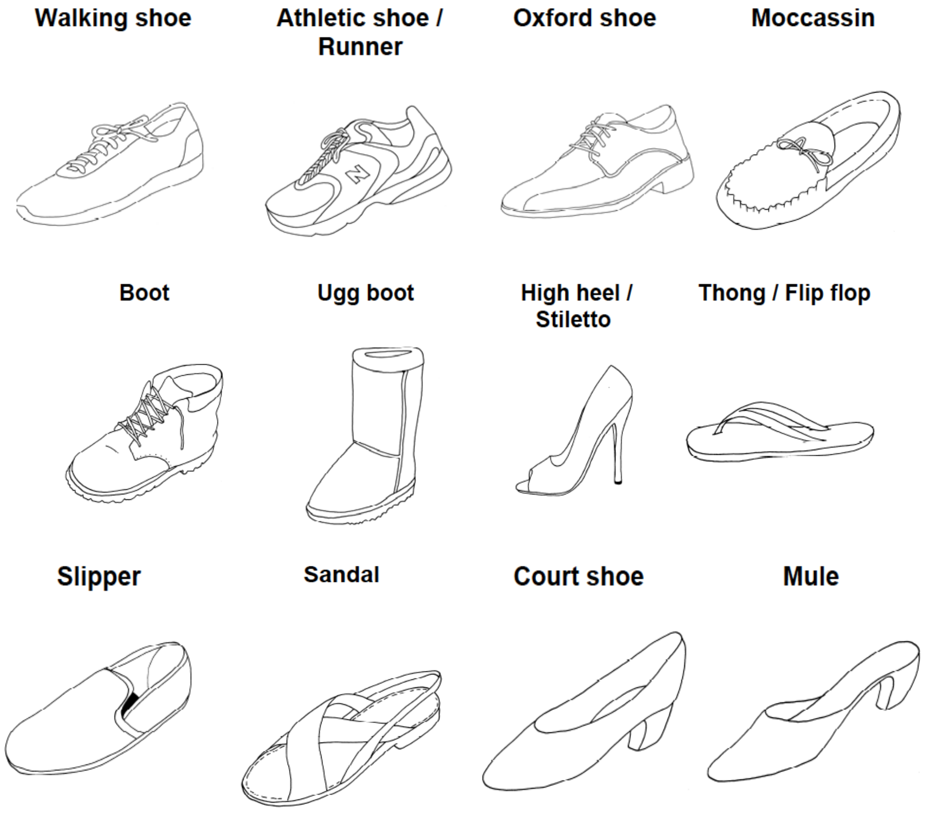

WEEK 1!

Please write Monday's date here: ____

Please record the following information each day over the week.

Daily Record	Monday	Tuesday	Wednesday	Thursday	Friday	Saturday	Sunday
What activities or sport did you do today?							
How was your knee pain today on a scale of 0-10?							
What type of shoes did you wear today? Please look at the examples on page 1.							
How many hours did you wear your shoe inserts today?							
Did you have any problems with your shoe inserts today?							
Did you take any medication for your knee pain today? If yes, please write down what you took (e.g. Panadol, Nurofen)							
Is there anything else you'd like to tell us about today?							

WEEK 2!

Please write Monday's date here: ____

Please record the following information each day over the week.

Daily Record	Monday	Tuesday	Wednesday	Thursday	Friday	Saturday	Sunday
What activities or sport did you do today?							
How was your knee pain today on a scale of 0-10?							
What type of shoes did you wear today? Please look at the examples on page 1.							
How many hours did you wear your shoe inserts today?							
Did you have any problems with your shoe inserts today?							
Did you take any medication for your knee pain today? If yes, please write down what you took (e.g. Panadol, Nurofen)							
Is there anything else you'd like to tell us about today?							

WEEK 3!

Please write Monday's date here: ____

Please record the following information each day over the week.

Daily Record	Monday	Tuesday	Wednesday	Thursday	Friday	Saturday	Sunday
What activities or sport did you do today?							
How was your knee pain today on a scale of 0-10?							
What type of shoes did you wear today? Please look at the examples on page 1.							
How many hours did you wear your shoe inserts today?							
Did you have any problems with your shoe inserts today?							
Did you take any medication for your knee pain today? If yes, please write down what you took (e.g. Panadol, Nurofen)							
Is there anything else you'd like to tell us about today?							

WEEK 4!

Please write Monday's date here: ____

Please record the following information each day over the week.

Daily Record	Monday	Tuesday	Wednesday	Thursday	Friday	Saturday	Sunday
What activities or sport did you do today?							
How was your knee pain today on a scale of 0-10?							
What type of shoes did you wear today? Please look at the examples on page 1.							
How many hours did you wear your shoe inserts today?							
Did you have any problems with your shoe inserts today?							
Did you take any medication for your knee pain today? If yes, please write down what you took (e.g. Panadol, Nurofen)							
Is there anything else you'd like to tell us about today?							

WEEK 5!

Please write Monday's date here: ____

Please record the following information each day over the week.

Daily Record	Monday	Tuesday	Wednesday	Thursday	Friday	Saturday	Sunday
What activities or sport did you do today?							
How was your knee pain today on a scale of 0-10?							
What type of shoes did you wear today? Please look at the examples on page 1.							
How many hours did you wear your shoe inserts today?							
Did you have any problems with your shoe inserts today?							
Did you take any medication for your knee pain today? If yes, please write down what you took (e.g. Panadol, Nurofen)							
Is there anything else you'd like to tell us about today?							

WEEK 6!

Please write Monday's date here: ____

Please record the following information each day over the week.

Daily Record	Monday	Tuesday	Wednesday	Thursday	Friday	Saturday	Sunday
What activities or sport did you do today?							
How was your knee pain today on a scale of 0-10?							
What type of shoes did you wear today? Please look at the examples on page 1.							
How many hours did you wear your shoe inserts today?							
Did you have any problems with your shoe inserts today?							
Did you take any medication for your knee pain today? If yes, please write down what you took (e.g. Panadol, Nurofen)							
Is there anything else you'd like to tell us about today?							

HAPPi KNEECAPS!

WEEK 7! Please write Monday's date here: ____

Please record the following information each day over the week.

Daily Record	Monday	Tuesday	Wednesday	Thursday	Friday	Saturday	Sunday
What activities or sport did you do today?							
How was your knee pain today on a scale of 0-10?							
What type of shoes did you wear today? Please look at the examples on page 1.							
How many hours did you wear your shoe inserts today?							
Did you have any problems with your shoe inserts today?							
Did you take any medication for your knee pain today? If yes, please write down what you took (e.g. Panadol, Nurofen)							
Is there anything else you'd like to tell us about today?							

WEEK 8!

Please write Monday's date here: ____

Please record the following information each day over the week.

Daily Record	Monday	Tuesday	Wednesday	Thursday	Friday	Saturday	Sunday
What activities or sport did you do today?							
How was your knee pain today on a scale of 0-10?							
What type of shoes did you wear today? Please look at the examples on page 1.							
How many hours did you wear your shoe inserts today?							
Did you have any problems with your shoe inserts today?							
Did you take any medication for your knee pain today? If yes, please write down what you took (e.g. Panadol, Nurofen)							
Is there anything else you'd like to tell us about today?							

WEEK 9!

Please write Monday's date here: ____

Please record the following information each day over the week.

Daily Record	Monday	Tuesday	Wednesday	Thursday	Friday	Saturday	Sunday
What activities or sport did you do today?							
How was your knee pain today on a scale of 0-10?							
What type of shoes did you wear today? Please look at the examples on page 1.							
How many hours did you wear your shoe inserts today?							
Did you have any problems with your shoe inserts today?							
Did you take any medication for your knee pain today? If yes, please write down what you took (e.g. Panadol, Nurofen)							
Is there anything else you'd like to tell us about today?							

WEEK 10!

Please write Monday’s date here: ____

Please record the following information each day over the week.

Daily Record	Monday	Tuesday	Wednesday	Thursday	Friday	Saturday	Sunday
What activities or sport did you do today?							
How was your knee pain today on a scale of 0-10?							
What type of shoes did you wear today? Please look at the examples on page 1.							
How many hours did you wear your shoe inserts today?							
Did you have any problems with your shoe inserts today?							
Did you take any medication for your knee pain today? If yes, please write down what you took (e.g. Panadol, Nurofen)							
Is there anything else you’d like to tell us about today?							

WEEK 11! Please write Monday's date here: ____

Please record the following information each day over the week.

Daily Record	Monday	Tuesday	Wednesday	Thursday	Friday	Saturday	Sunday
What activities or sport did you do today?							
How was your knee pain today on a scale of 0-10?							
What type of shoes did you wear today? Please look at the examples on page 1.							
How many hours did you wear your shoe inserts today?							
Did you have any problems with your shoe inserts today?							
Did you take any medication for your knee pain today? If yes, please write down what you took (e.g. Panadol, Nurofen)							
Is there anything else you'd like to tell us about today?							

WEEK 12!

Please write Monday's date here: ______________________________

Please record the following information each day over the week.

Daily Record	Monday	Tuesday	Wednesday	Thursday	Friday	Saturday	Sunday
What activities or sport did you do today?							
How was your knee pain today on a scale of 0-10?							
What type of shoes did you wear today? Please look at the examples on page 1.							
How many hours did you wear your shoe inserts today?							
Did you have any problems with your shoe inserts today?							
Did you take any medication for your knee pain today? If yes, please write down what you took (e.g. Panadol, Nurofen)							
Is there anything else you'd like to tell us about today?							

Please write any other comments here:
